# Supplementary material for: Low Stress Ion Conductance Microscopy of Sub-Cellular Stiffness
Source: Soft Matter. 2016 Aug 17;12(38):7953–8. doi: 10.1039/c6sm01106c (PMC5166566; doi:10.1039/c6sm01106c)
Supplement: Supplementary file 1 [file SM-012-C6SM01106C-s001.pdf]

Electronic Supplementary Material (ESI) for Soft Matter.
This journal is © The Royal Society of Chemistry 2016

Soft Matter

Electronic Supplementary Information

Low Stress Ion Conductance Microscopy of Sub-Cellular Stiffness

Electronic Supplementary Information

Richard W. Clarke,‡*[a] Pavel Novak,‡[b] Alexander Zhukov,[a] Eleanor J. Tyler,[c] Marife Cano-Jaimez,[d] Anna Drews,[a] Owen Richards,[a] Kirill Volynski,[d] Cleo Bishop[c] and David Klenerman*[a]

www.rsc.org/

CONTENTS

Supplementary Methods

- Apparatus
- Computation
- Materials
- Cells

Expanded data presentations of

- Approach curves
- Stiffness maps
- Nanopipet stresses and forces

References

[a] University Chemical Laboratories, Lensfield Road, Cambridge, CB2 1EW.
[b] School of Engineering and Materials Science, Queen Mary University of London, Mile End Road, London, E1 4NS.
[c] Centre for Cell Biology and Cutaneous Research, Queen Mary University of London, 4 Newark Street, London, E1 2AT.
[d] UCL Institute of Neurology, Queen Square, London, WC1N 3BG.
‡ These authors contributed equally.
* Correspondence to: rwc25@cam.ac.uk; dk10012@cam.ac.uk.

Supplementary Methods

Apparatus

We used ICM instruments based in London, described previously,[1] and Cambridge. The Cambridge instrument, based on an Eclipse Ti-U inverted microscope (Nikon), was built on a MK26 vibration isolation table (Minus k) and enclosed inside a Faraday cage. The stage was stabilized by a kinematic mount from an aluminium top-plate to a 12 mm M-461-XY-M stage (Newport) fixed to an aluminium underplate and adjusted by micrometers to align the optical axis. Samples are positioned by two DM-13L lockable differential micrometers (Newport) mounted horizontally on the underplate at 45 degrees to the microscope eyepiece, tensioned by steel tensators to hardened steel stops on an aluminium block holding a 30 µm XY P-733.2DD piezoelectric drive (PI) with a plastic push-in dish mount for 35 mm dishes (Corning). Nanopipets are clamped vertically into a v-groove on an acrylic pipette holder on a 25 µm P-753 piezoelectric drive (PI) mounted below the current amplifier headstage (Axopatch 200B, Molecular Devices), both held to a 25mm M-462-X-M crossed-roller bearing translation stage (Newport) driven by a lead-screw 25mm M-112.1DG motor (PI), kept to one extreme of yaw by a tensator. Pairs of nanopipets were made by a P-2000 puller (Sutter), pulling 10 cm long, 0.5/1.0 mm inner/outer diameter fire-polished borosilicate glass capillaries with filaments (Intracel). With the program Heat 350, Fil 3, Vel 30, Del 220, Pul 0, Heat 390, Fil 3, Vel 40, Del 180, Pul 255 we made small, 400-200 MΩ, nanopipets. Half of these were already in the target range of 300-100 MΩ; a 50% increase in current was made as needed by flattening the tip face against the sample dish at 100 $nm.ms^{-1}$ whilst ensuring the nanopipet rapidly rose at the first sign of any current decrease. Larger, typically 125-80 MΩ nanopipets were made with the program Heat 310, Fil 3, Vel 30, Del 160, Pul 0, Heat 330, Fil 3, Vel 25, Del 160, Pul 200. All experiments applied 200 mV and used physiological solutions, of conductivity 1.35 $S.m^{-1}$. Actuators were commanded by customized hopping mode[2, 3] software on digital controllers (Ionscope). The scanning control sequence ascertains the ion current in bulk solution while shifting the horizontal sample position, then lowers the nanopipet to register the heights at which the ion current decreases by set fractions, typically 0.3% and 2%, before repeating the cycle. The approach control sequence records ion current at many heights as the nanopipet is lowered. Approaches to HpL cells also alternated the changes in height between increases and decreases, verifying that the cells responded elastically in the ranges assayed.

Computation

All curves are computed parametrically in terms of the tip-surface gap and the graphs of intrinsic and direct stress show the full range – many cell types would be fully compressed at lower total stresses than these curves reach. The force curves are calculated by multiplying the stress by the area of the tip face, evaluated from the conserved ratio of wall thickness to inner diameter in nanopipets pulled from glass capillaries. The parametric equations for fitting approach curves to cells described in the main text may be summarised in terms of the functions defined there as $[z, I] = [h(\sigma(g) + \varsigma(g)) + g, I(g)]$ in general and for neuronal cells as $[z, I] = [h_{(C)}(\sigma(g)) + h_{(S)}(\sigma(g)) + g, I(g)]$. Images of topographic and stiffness maps were rendered using the Gwyddion scanning probe microscopy software suite.[4] The fits in Figs. 2 and S2 are representative of 16, 37 and 11 approaches for HN, HpL, and HMF cells respectively. Fourteen of the sixteen HN approaches required the dual stiffness model; the other two must have assayed points on the cell surface that were directly attached to the cytoskeleton. To avoid singularities in stiffness maps including substrate we limit gap-corrected height difference fields to a non-zero minimum of 1 nm. The HN maps in Figs. 3, S3 & S4 are representative of eleven scans, two of which were non-independent zooms not repeated in the averages quoted in the main text. The highest parts of these cells were pushed down by around 200 nm. The stiffness maps of HpL cells in Figs. 3 and S5 are a special case where the pairs of height measurements were not acquired during a single scan but were instead determined from two consecutive scans. We linearly aligned these pairs of topographies by reference to the substrate which in these circumstances already accounts for the difference in pipette-cell gap and slight thermal drift. However, in between the two scans for Fig. 3(c) the cell actively moved upwards in a few places, creating point artifacts of high stiffness. In order to prevent these from impairing contrast we have limited the stiffness scale of this particular map to three times the root mean square value. This issue does not affect any of the other stiffness maps, whose pairs of height measurements were acquired in rapid succession at each point of single scans, an improved method. The two HpL maps in Fig. S5 are independent scans; the absolute height of the substrate in the right-hand panel was determined by reference to an earlier scan. The HMF maps in Figs. S6 & S7 are representative of seven scans, three of which were non-independent zooms not repeated in the averages quoted in the main text. Due to noise in the ion current some scans have two or three outlying height measurements that rise more than 1 µm above the surrounding topography. To improve contrast it is routine in ICM imaging to replace these spikes with averages of the surrounding heights, a minor correction as illustrated by the similarity of Figs. 3(f) and S6. For the purpose of illustration the latter topography is left uncorrected for the effect of active cell movement during the scan, which creates mismatches between the bands of measurement points. Where discernible, we minimize these topographic mismatches by piece-wise linear regression of the stripe differences, retaining the original fields of measured height differences for the stiffness map calculations.

Materials

All chemicals were reagent grade and used as supplied. Solutions were filtered by Anotop 0.02 µm filters (Whatman). All measurements were performed in polystyrene dishes (Corning), unless otherwise noted.

Cells

Hippocampal neurons: HN cells were cultured in Neurobasal A/B27 based medium on an astrocyte feeder layer plated on 19 mm glass coverslips covered with poly-D-lysine[5] having been isolated from humanely killed P0–P2 rats.[6] The standard extracellular solution used in experiments on hippocampal neurons contained 125 mM NaCl, 2.5 mM KCl, 2 mM MgCl2, 2 mM CaCl2, 30 mM glucose, 0.01 mM NBQX, 0.05 mM APV, and 25 mM HEPES (pH 7.4). HpL neuronal cells: The HpL cell line is derived from mouse hippocampal neurons transformed by SV40 [7] and is also known as P4. These cells were grown in 75 cm^2 flasks with OptiMEM (Invitrogen) plus 10% fetal bovine serum, 0.5% penicillin, 0.5% streptomycin in humid air at 37˚C, 5% CO2. For ICM experiments the cells were cultured to 70–90% confluence in 35 mm culture dishes (Corning) with 14 mm glass microwell, number 1 thickness cover glass (MatTek). Scans were conducted in Leibovitz-15 (L15) medium (Invitrogen), 1-2 days after plating. Mammary fibroblasts: Normal human mammary fibroblasts were cultured as previously described[8] with L15 medium as the bath solution for ICM experiments.

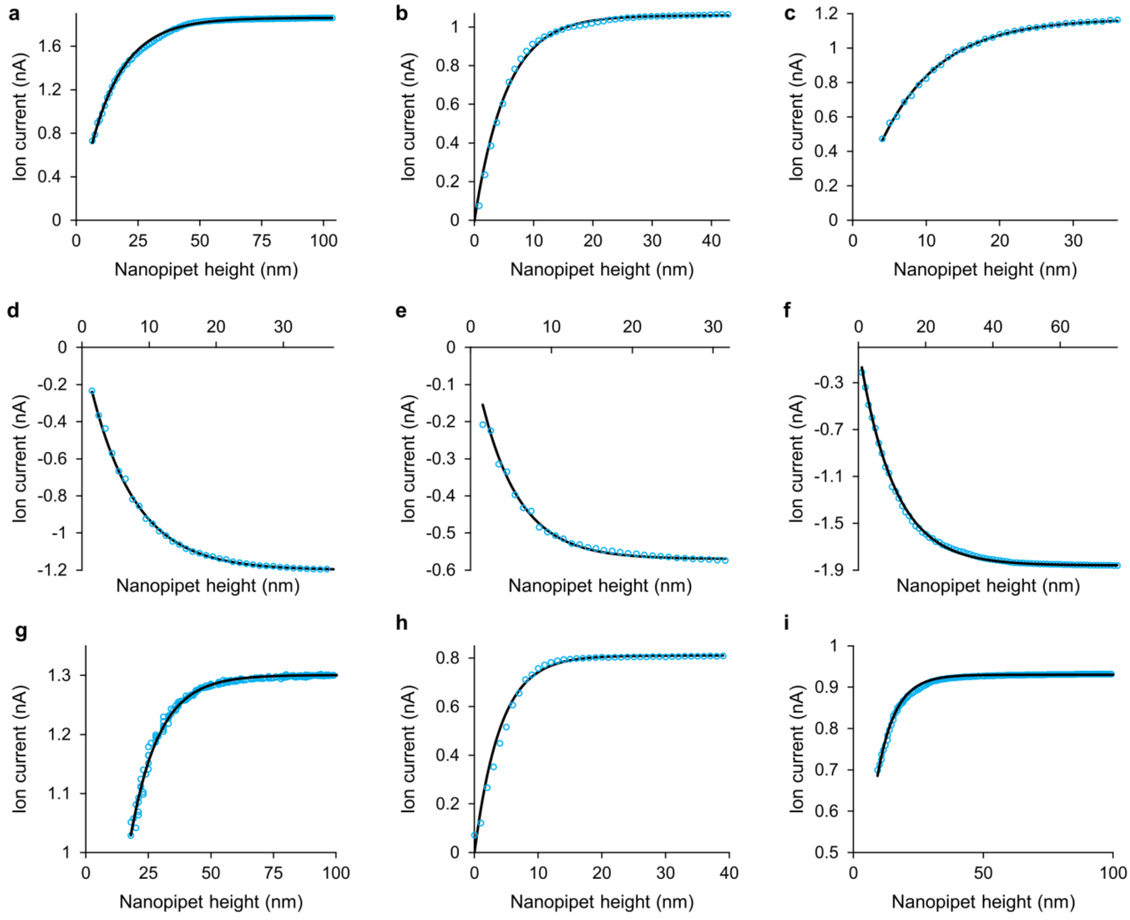

Figure S1. Approach calibrations. (**a**) 84 nm aperture (108 MΩ) nanopipet, fitting x=3.1. (**b**) 48 nm aperture (189 MΩ) nanopipet, fitting x=4.4. (**c**) 53 nm aperture (171 MΩ) nanopipet, fitting x=3.3. (**d**) 54 nm aperture (167 MΩ) nanopipet, fitting x=4. (**e**) 26 nm aperture (351 MΩ) nanopipet, fitting x=2.7. (**f**) 84 nm aperture (108 MΩ) nanopipet, fitting x=4. (**g**) 59 nm aperture (154 MΩ) nanopipet, fitting x=2.55. (**h**) 36 nm aperture (247 MΩ) nanopipet, fitting x=4.5. (**i**) 42 nm aperture (215 MΩ) nanopipet, fitting x=3. Mean geometric factor x=3.6±0.2 (N=19).

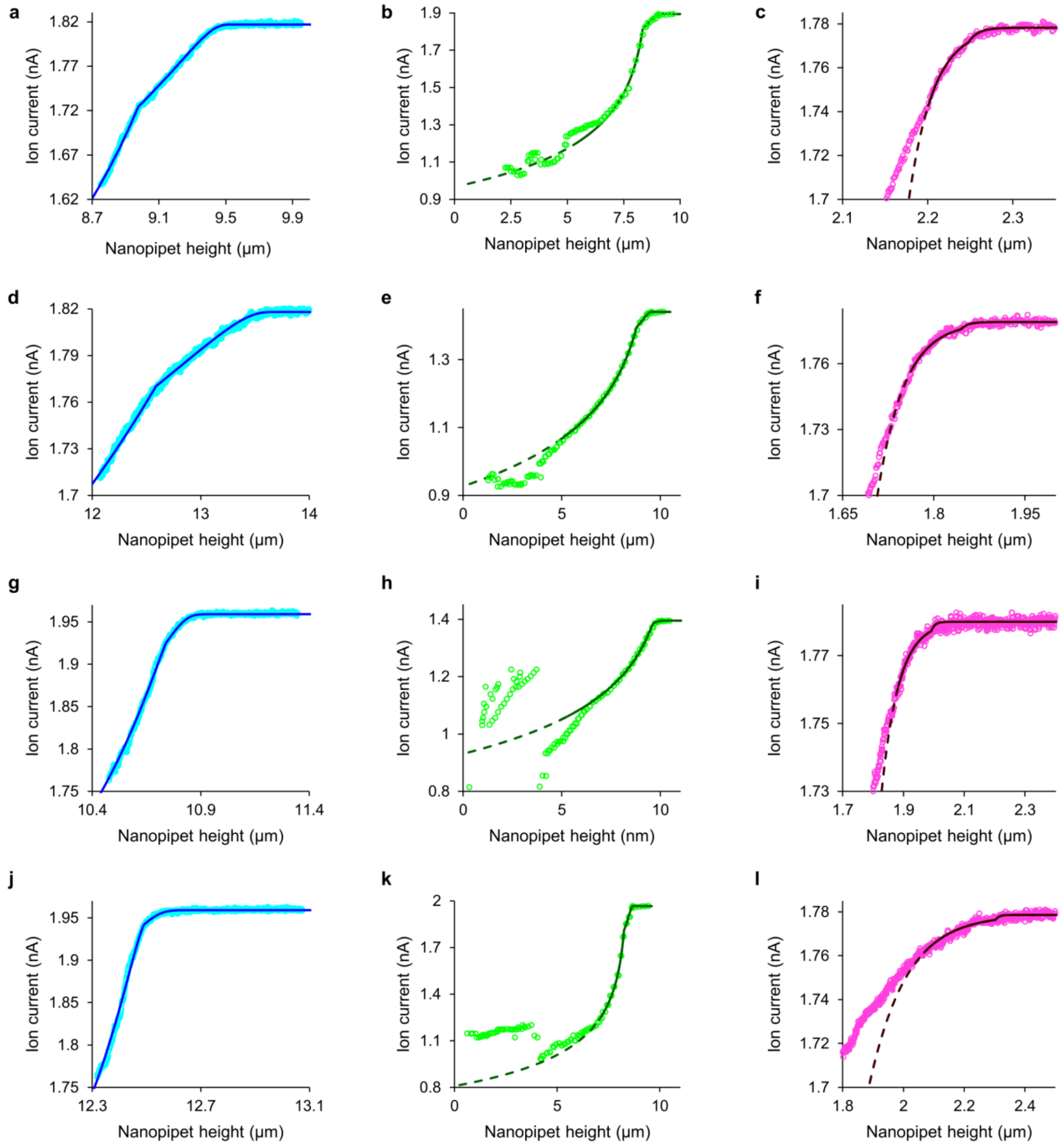

Figure S2. Cell approaches. (**a**) HN, apparent stiffness 107 Pa for 483 nm, fits 260 Pa cortex 9.135 µm thick with 5.5 Pa, 290 nm slack. (**b**) HpL, apparent stiffness 51.7 Pa for 0.7 µm, fits 350 Pa, 8.4 µm cortex with 4 Pa, 0.6 µm slack. (**c**) HMF fits 9.0 kPa, 2.2 µm with 62 nm glycocalyx. (**d**) HN, apparently 40.4 Pa for 1.01 µm, fits 81 Pa, 13.03 µm cortex with 3 Pa, 525 nm slack. (**e**) HpL, apparently 90.1 Pa for 738 nm, fits 265 Pa, 9 µm cortex with 7 Pa, 0.5 µm slack. (**f**) HMF fits 3.5 kPa, 1.775 µm with 70 nm glycocalyx. (**g**) HN, apparently 171 Pa for 111 nm, fits 337 Pa, 10.75 µm cortex with 1.75 Pa, 55 nm slack. (**h**) HpL, apparently 50.4 Pa for 0.5 µm, fits 240 Pa, 9.7 µm cortex with 2.5 Pa, 0.4 µm slack. (**i**) HMF, 2.6 kPa, 1.92 µm with 72 nm glycocalyx. (**j**) HN, apparently 175 Pa for 78 nm, fits 760 Pa, 12.45 µm cortex with 1.09 Pa, 60 nm slack. (**k**) HpL, apparently 160 Pa for 375 nm, fits 770 Pa, 8.3 µm cortex with 7 Pa, 0.3 µm slack. (**l**) HMF, 1.25 kPa, 2.23 µm with 74 nm glycocalyx.

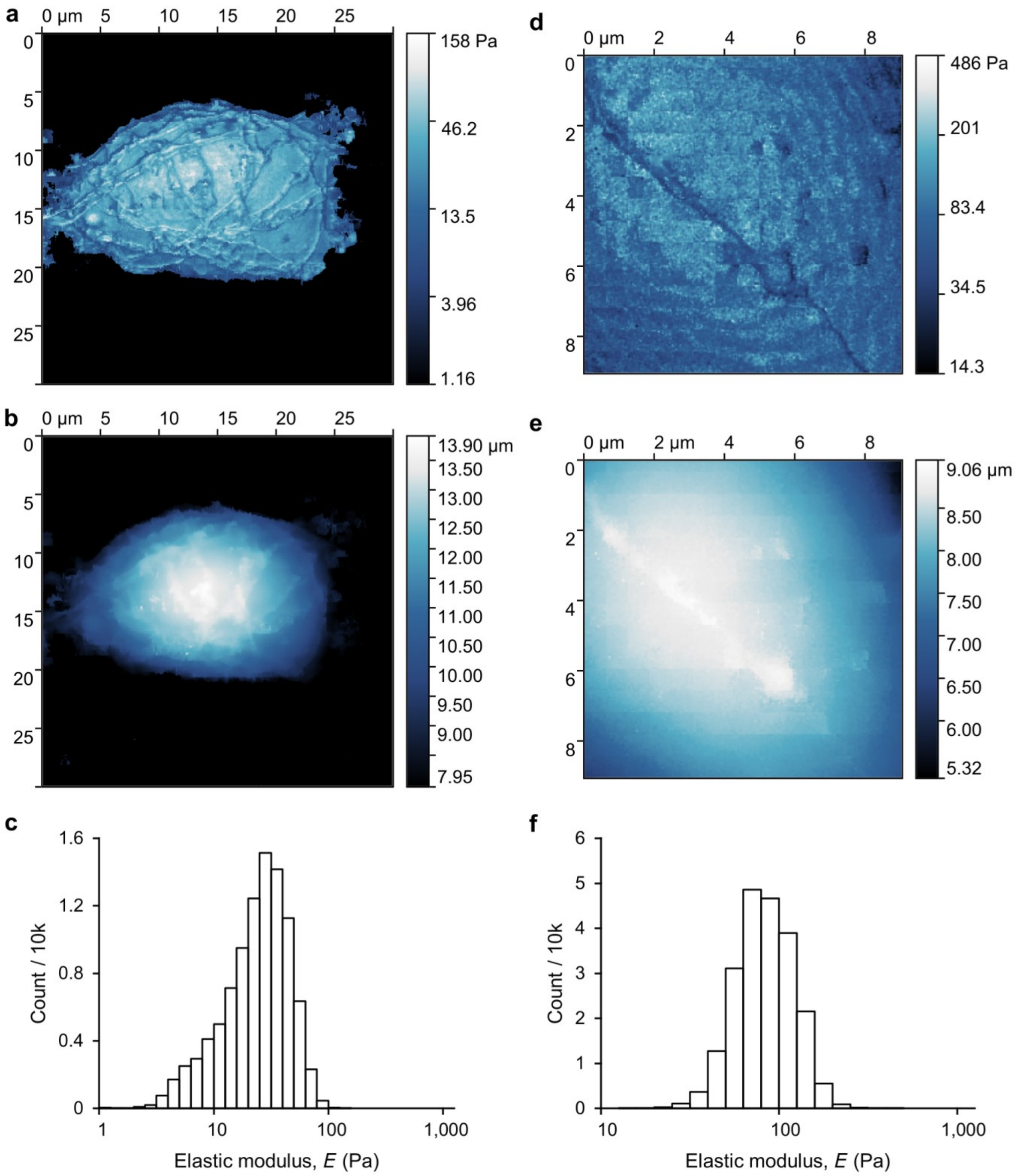

Figure S3. Maps of hippocampal neuron cell stiffness and topography. (**a**) Cell body stiffness, selected as the top 50% topography, mapped with an 81 MΩ nanopipet of 110 nm aperture at ΔI=0.3%, 2%, exerting stress of 0.29 Pa, 0.96 Pa at gaps of 90 nm, 60 nm respectively. (**b**) Top 50% topography. (**c**) Histogram of cell body stiffness, mean 28 Pa. (**d**) Higher resolution map of the top of the cell body of a hippocampal neuron, 9.063 µm by 9.063 µm, mapped with a 145 MΩ nanopipet of 62 nm aperture at ΔI=0.5%, 1%, exerting stress of 2.23 Pa, 3.40 Pa at gaps of 46 nm, 40 nm respectively. (**e**) Topography. (**f**) Stiffness histogram, mean 89 Pa.

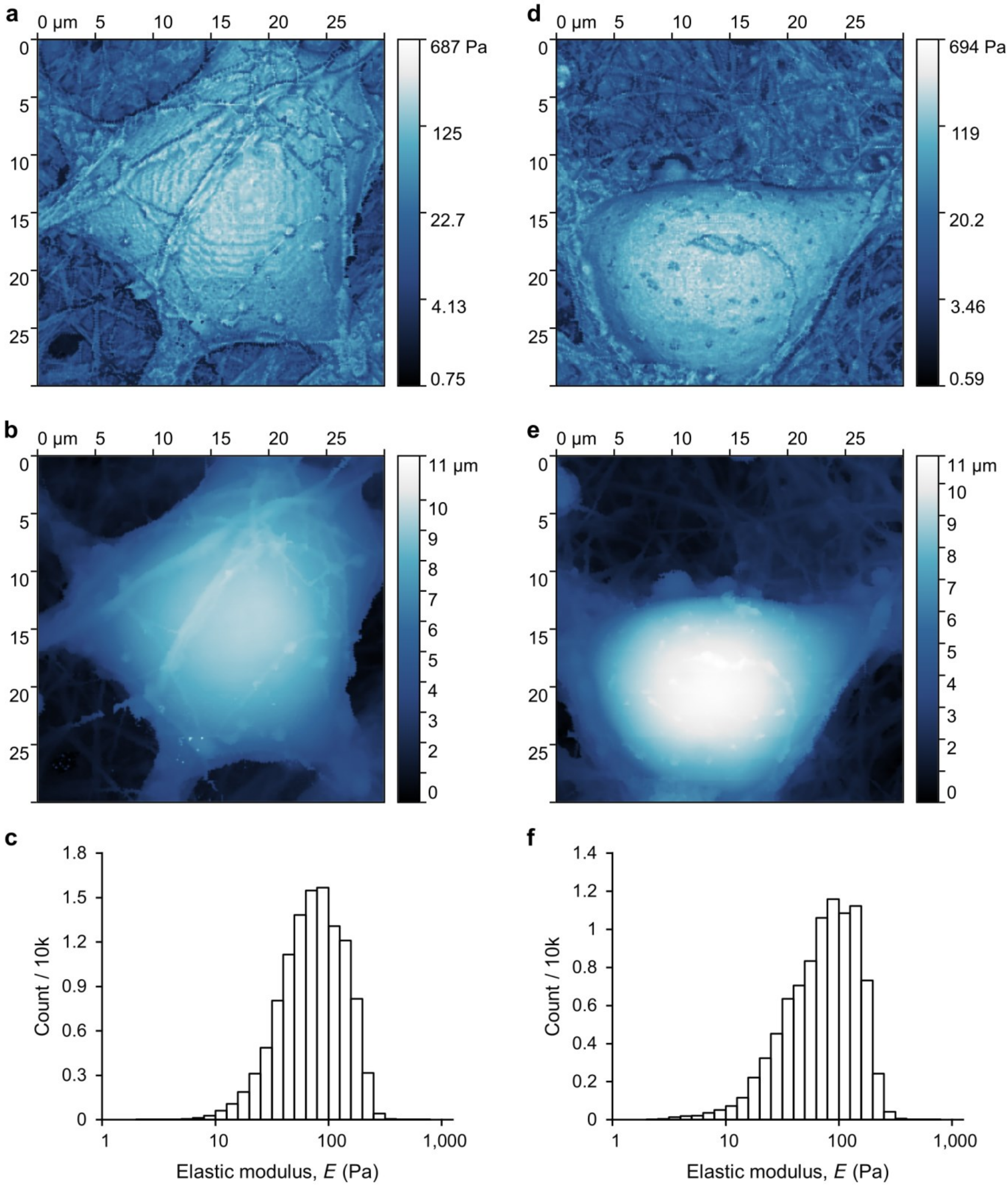

Figure S4. Maps of hippocampal neuron cell stiffness and topography. (**a**) Cell stiffness mapped with a 110 MΩ nanopipet of 81.8 nm aperture diameter at ΔI=0.3%, 1%, exerting stress of 0.74 Pa, 1.48 Pa at gaps of 66 nm, 52 nm respectively. (**b**) Topography. (**c**) Histogram of cell body stiffness, selected as top 50% topography; mean 86.6 Pa. (**d**) Cell stiffness mapped with a 102 MΩ nanopipet of 88.2 nm aperture diameter at ΔI=0.3%, 1.5%, exerting stress of 0.59 Pa, 1.56 Pa at gaps of 71 nm and 51 nm respectively. (**e**) Topography. (**f**) Cell body stiffness, selected as top 50% topography; mean 87.5 Pa.

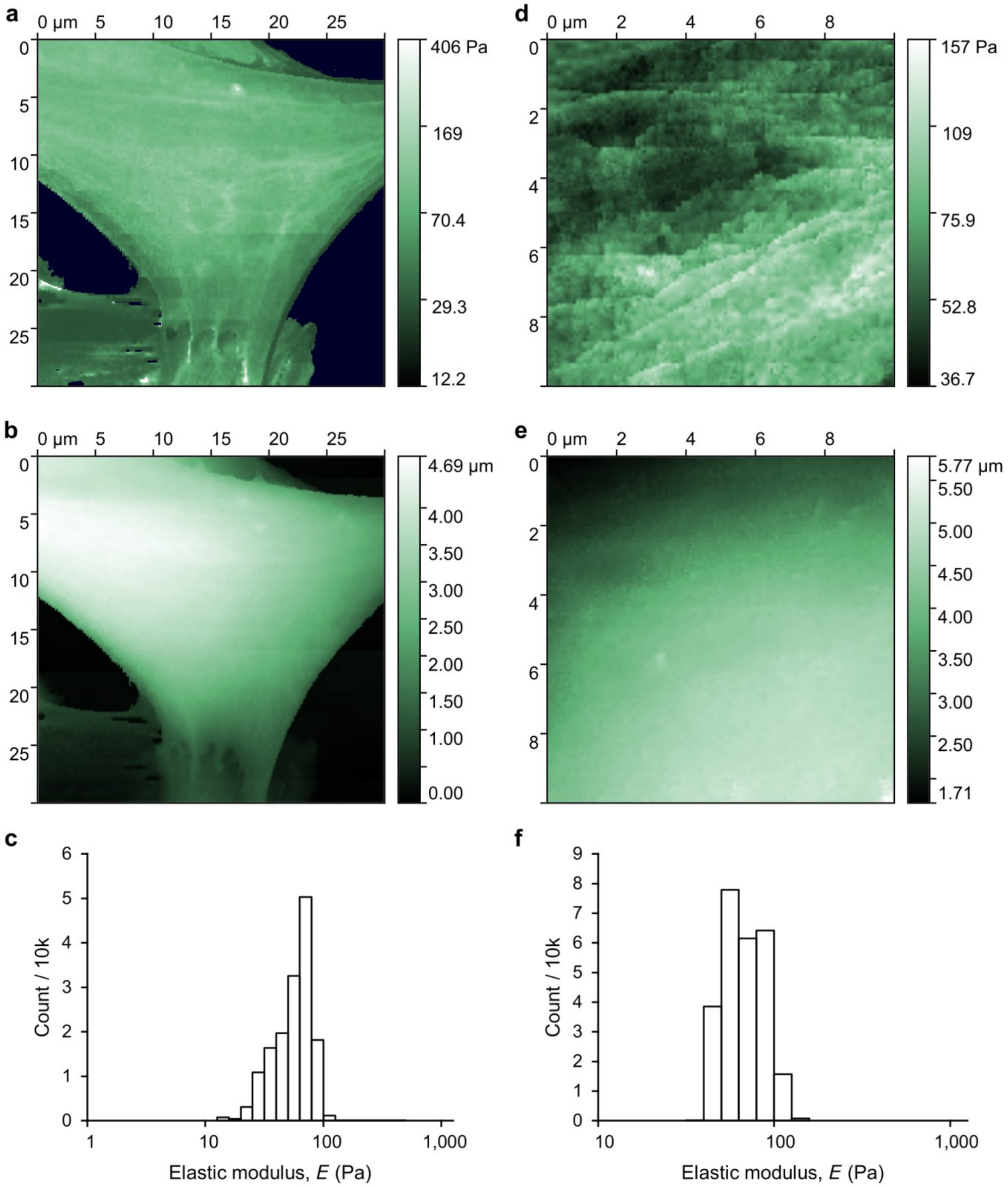

Figure S5. Maps of HpL neuronal cell stiffness and topography. (**a**) Cell stiffness mapped with a 174 MΩ nanopipet of 51.8 nm aperture diameter at ΔI=0.6%, 3%. This exerts stress of 4.27 Pa, 13.26 Pa at gaps of 37 nm, 25 nm. (**b**) Topography. (**c**) Histogram of cell body stiffness, selected as top 70% topography; mean 58.7 Pa. (**d**) Higher resolution map of the stiffness of a 10 μm by 10 μm area on top of a different cell mapped with the same nanopipet at ΔI=0.6%, 4.2%, exerting stress of 4.27 Pa, 17.95 Pa at gaps of 37 nm, 23 nm. (**e**) Topography. (**f**) Stiffness histogram, mean 70 Pa.

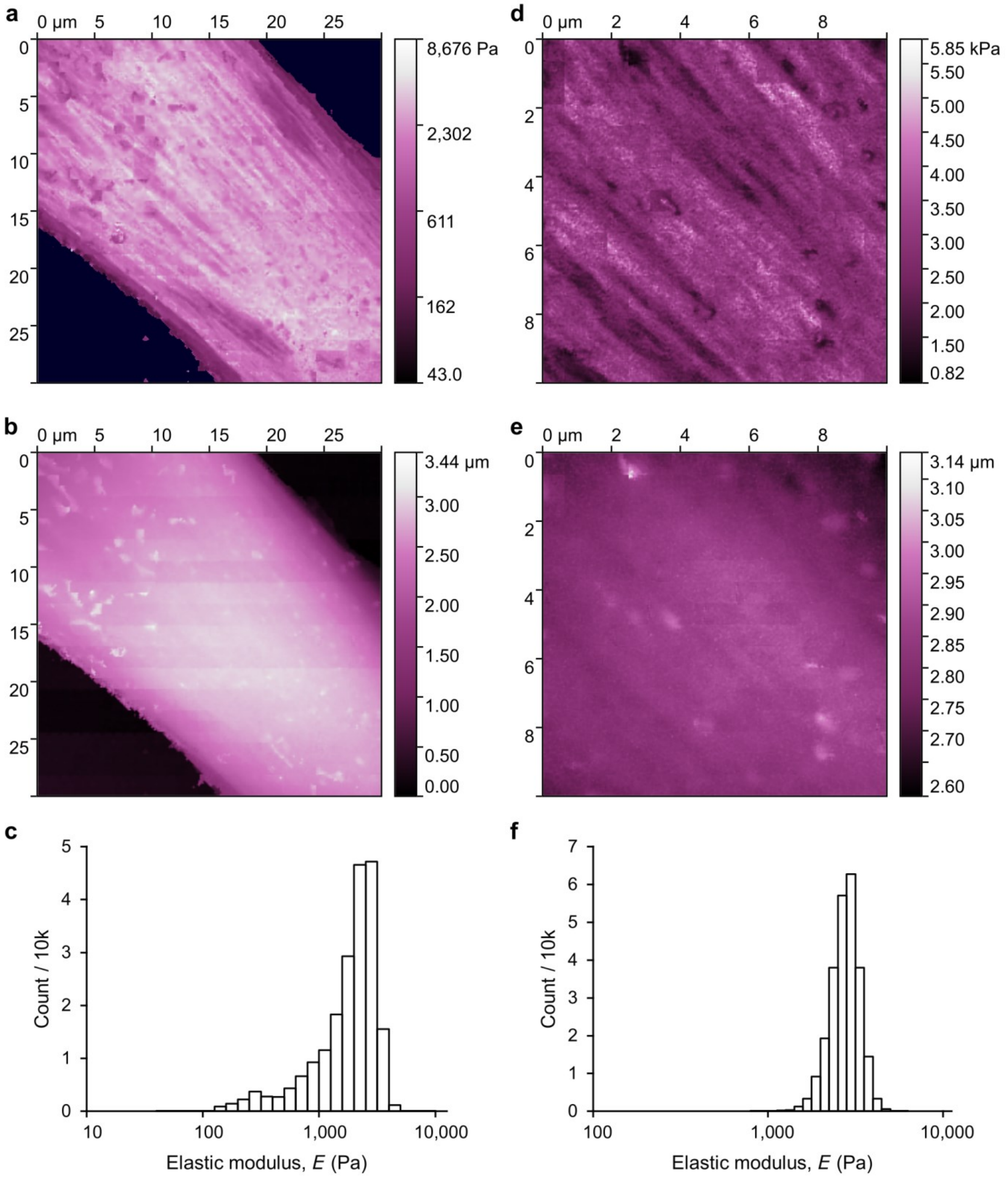

Figure S6. Maps of mammary fibroblast cell stiffness and topography. (**a**) Cell stiffness mapped with a 90 MΩ nanopipet of 100 nm aperture diameter at ΔI=0.3%, 3.0%, exerting stress of 0.4 Pa, 122.5 Pa at gaps of 81 nm, 49 nm. (**b**) Topography. For comparison this topography is not corrected for active cell movement during the data acquisition as detailed in methods. (**c**) Histogram of cell stiffness, selected by top 90% topography; mean 1.992 kPa, mode 2.506 kPa. (**d**) Higher resolution map of the stiffness of a 10 μm by 10 μm area on the top of the same cell using the same stress levels. (**e**) Topography. (**f**) Histogram of stiffness measurements, mean 2.792 kPa.

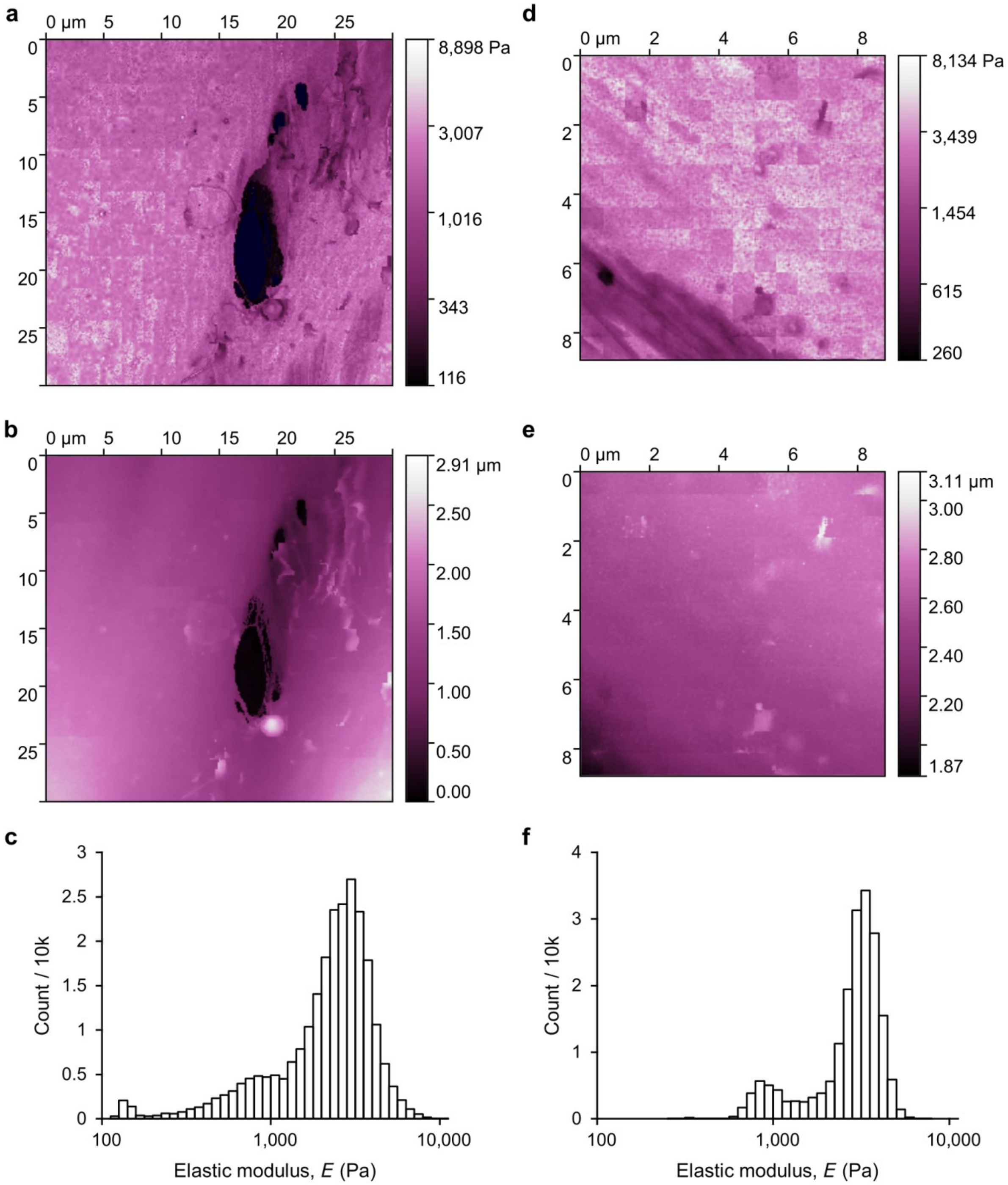

Figure S7. Maps of mammary fibroblast cell stiffness and topography. (**a**) Cell stiffness mapped with a 112 MΩ nanopipet of 80 nm aperture diameter at ΔI=0.3%, 2.0%, exerting stress of 33.4 Pa, 151.9 Pa at gaps of 64.6 nm, 43.5 nm. (**b**) Topography. (**c**) Histogram of cell stiffness, selected as top 90% topography; mean 2.44 kPa, mode 2.82 kPa. (**d**) Higher resolution map of the stiffness of an 8.79 μm square area on a different cell mapped with a 90 MΩ nanopipet of 100 nm aperture diameter at ΔI=0.3%, 3.0%, exerting stress of 0.4 Pa, 122.5 Pa at gaps of 81 nm, 49 nm. (**e**) Topography. (**f**) Histogram of stiffnesses, mean 2.89 kPa.

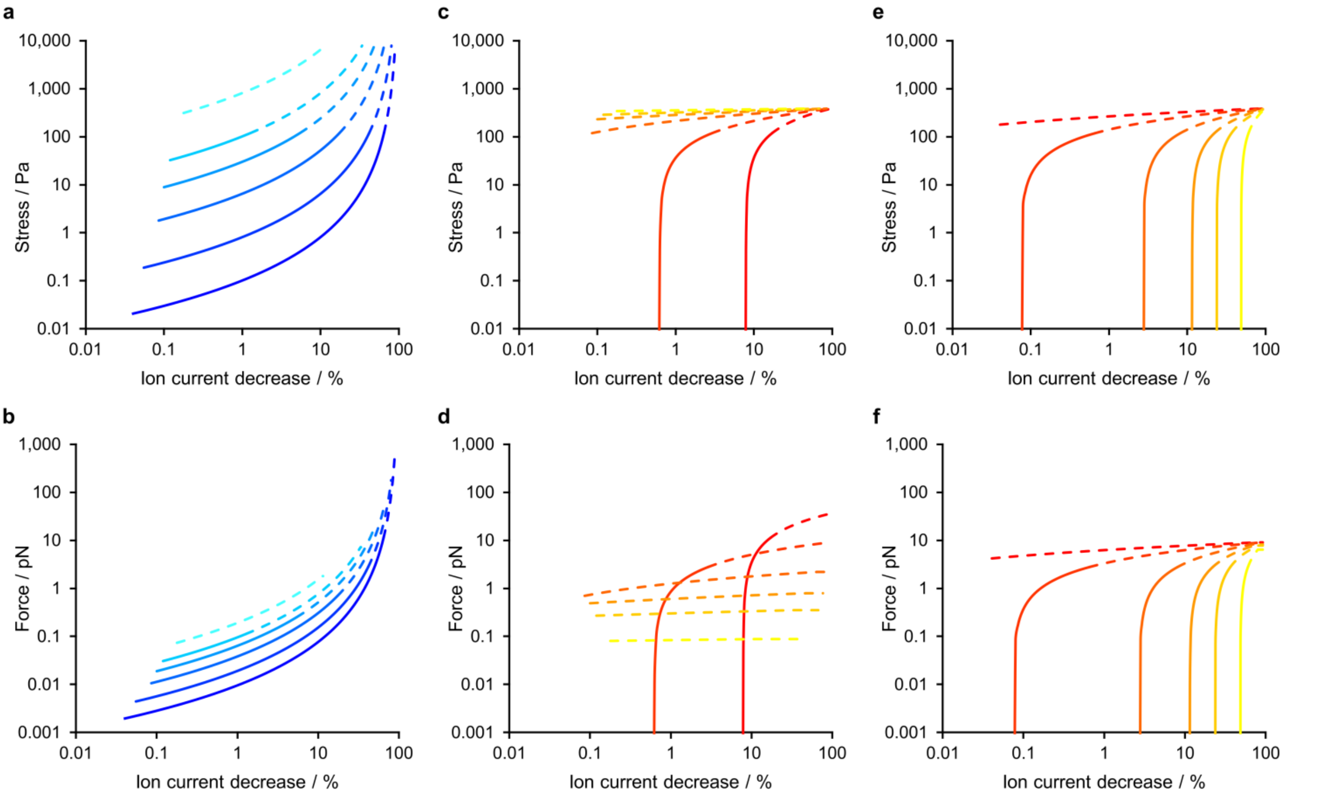

Figure S8. Stresses and forces versus ion current decrease calculated for ranges of aperture diameter and glycocalyx thickness. (**a**) Intrinsic stress for nanopipets of aperture diameters 10, 20, 30, 50, 100 and 200 nm, shown cyan to blue. The lines start at the minimum detectable ion current decrease in 1 ms and the dashes run from the bleb stress to the joining pressure. (**b**) Intrinsic force. Larger pipettes can apply a wider range of force before detaching any membrane because they detect the cell from further away and have larger tip-face area. (**c**) Direct stress on glycocalyx 70.5 nm thick for nanopipets of the same inner diameters, shown yellow to red. The smaller nanopipets would push the glycocalyx before a usable current decrease of 0.1%. (**d**) Direct force on this thickness of glycocalyx. (**e**) Direct stress exerted by a 100 nm aperture nanopipet for glycocalyx thicknesses of 10, 20, 30, 50, 100 and 200 nm, shown yellow to red. (**f**) Direct force on these thicknesses of glycocalyx; the asymptotes are equal here because the tip face areas are the same.

References

1. P. Novak, J. Gorelik, U. Vivekananda, A. I. Shevchuk, Y. S. Ermolyuk, R. J. Bailey, A. J. Bushby, G. W. Moss, D. A. Rusakov and D. Klenerman, *Neuron*, 2013, **79**, 1067-1077.
2. P. Novak, C. Li, A. I. Shevchuk, R. Stepanyan, M. Caldwell, S. Hughes, T. G. Smart, J. Gorelik, V. P. Ostanin and M. J. Lab, *Nature methods*, 2009, **6**, 279-281.
3. P. Happel and I. D. Dietzel, *Journal of nanobiotechnology*, 2009, **7**, 1.
4. D. Nečas and P. Klapetek, *Open Physics*, 2012, **10**, 181-188.
5. Y. S. Ermolyuk, F. G. Alder, C. Henneberger, D. A. Rusakov, D. M. Kullmann and K. E. Volynski, 2012.
6. J. Waters and S. J. Smith, *The Journal of physiology*, 2002, **541**, 811-823.
7. C. Kuwahara, A. M. Takeuchi, T. Nishimura, K. Haraguchi, A. Kubosaki, Y. Matsumoto, K. Saeki, Y. Matsumoto, T. Yokoyama and S. Itohara, *Nature*, 1999, **400**, 225-226.
8. S. R. Romanov, B. K. Kozakiewicz, C. R. Holst, M. R. Stampfer, L. M. Haupt and T. D. Tlsty, *Nature*, 2001, **409**, 633-637.
